# Rangewide ploidy variation and evolution in *Acacia senegal*: a north–south divide?

**DOI:** 10.1093/aobpla/plv011

**Published:** 2015-02-13

**Authors:** David W. Odee, Julia Wilson, Stephen Omondi, Annika Perry, Stephen Cavers

**Affiliations:** 1 Kenya Forestry Research Institute, PO Box 20412-00200, Nairobi, Kenya; 2 Centre for Ecology and Hydrology, Bush Estate, Penicuik, Midlothian EH26 0QB, UK

**Keywords:** *Acacia senegal*, African acacias, diploids, DNA ploidy level, dried leaves and twigs, flow cytometry, hexaploids, polyploidy, tetraploids, triploids

## Abstract

Knowledge of rangewide variation in DNA content and ploidy level may be valuable in understanding the evolutionary history of a species. Recent studies of *Acacia senegal* report diploids and occasional tetraploids in the Sudano-Sahelian region of sub-Saharan Africa, but nothing is known about the overall extent of DNA ploidy variation within the species. In this study, we determine the DNA content and ploidy level of *A. senegal* across its native range, and explore whether the variation is related to its evolutionary and colonization history. We used propidium iodide flow cytometry (FCM) to estimate DNA content (2C value) and infer ploidy in 157 individuals from 54 populations on various tissues, using seeds, fresh leaves, dried leaves and twigs and herbarium specimens. The mean 2C DNA (pg ± s.d.) contents detected were 1.47 ± 0.09, 2.12 ± 0.02, 2.89 ± 0.12, and a single individual with 4.51 pg, corresponding to a polyploid series of diploid, triploid, tetraploid and hexaploid individuals. Diploids were confirmed by chromosome counts (2*n* = 2*x* = 26). Most populations (90.7 %) were of single ploidy level, while mixed ploidy populations (9.3 %) comprising mostly diploids (2*x*+3*x*, 2*x*+4*x* and 2*x*+6*x*) were restricted to the Sudano-Sahelian and Indian subcontinent regions, its northern range. The species is predominantly diploid, and no mixed ploidy populations were detected in east and southern Africa, its southern range. The geographic pattern of ploidy variation in conjunction with existing phylogeographic and phylogenetic data of the species suggests that polyploids have occurred multiple times in its evolutionary and recent colonization history, including contemporary ecological timescales. The successful use of external tissues of dried twigs in FCM is new, and presents the opportunity to study numerous other dryland woody species.

## Abstract (precis)

A recent study of *Acacia senegal* showed a geographic pattern of genetic variation, which differentiated East and Southern African populations from those in the Sudano-Sahelian region. We build on this previous research to explore variation in DNA content using the flow cytometry method and chromosome number. A geographic north-south DNA content pattern was detected, reflecting the previous results. These results suggest that DNA content may also be important in elucidating the evolutionary history and distribution of the species. Use of external tissues of dried twigs in flow cytometry is new, and presents the opportunity to study numerous other dryland woody species.

## Introduction

Polyploidy, also known as whole-genome duplication, is widely recognized as a major driver of evolution, diversification and speciation in angiosperms (Soltis *et al*. 2009; Wood *et al*. 2009; Robertson *et al*. 2010). Cytological, fossil and genomic studies suggest that polyploidy is ancient and widespread, with most angiosperms estimated to have undergone one or more polyploidization events during their evolutionary history (Cui *et al*. 2006; Soltis *et al*. 2009; Wood *et al*. 2009; Jiao *et al*. 2011). Further analyses of cytogenetic and phylogenetic databases indicate that up to 15 % of angiosperm speciation events are accompanied by ploidy increase (Otto and Whitton 2000; Wood *et al*. 2009), although this number is likely to grow as more species are studied.

The establishment and maintenance of polyploids will depend on a range of complex ecological and reproductive processes that promote reproductive and/or spatial isolation between the newly formed polyploid plants and their progenitors (Thompson and Lumaret 1992; Petit *et al*. 1999; Baack 2004). Following genome multiplication, novel genetic combinations arise that may confer polyploids with different reproductive, morphological, ecological, physiological or cytological characteristics from their progenitors, allowing them to exploit new environments (Kolář *et al*. 2009; Manzaneda *et al*. 2012; Hao *et al*. 2013).

Variations in 2C DNA content and ploidy levels are not uncommon among closely related species, and can also be useful in delimiting taxonomically complex species (e.g. Kolář *et al*. 2009; Peirson *et al*. 2012). Furthermore, analysing 1Cx (monoploid) DNA may also be useful in comparing diploid–polyploid complexes across the natural range of the species; several workers have used the constancy in monoploid DNA as evidence of autopolyploidy (Balao *et al*. 2009; Duchoslav *et al*. 2013; Krejčíková *et al*. 2013). In many instances, polyploids and their diploid progenitors may co-occur across geographic regions referred to as contact zones, which can be useful in understanding the processes involved in their origins and evolutionary history (Husband and Schemske 1998; Petit *et al*. 1999). Diploid–polyploid contact zones may occur in two ways, namely (i) a primary contact zone, arising from the emergence of a new ploidy level within a diploid population, and (ii) a secondary contact zone, formed following contact between diploid and polyploid populations that were geographically isolated and have probably differentiated in allopatry (Petit *et al*. 1999). Several studies show that contact zones may either be localized or span wide geographic distances, and the dynamics of diploid–polyploid contact zones vary with species or species complexes (e.g. *Ranunculus adoneus*, Baack 2004; *Melampodium* spp., Steussy *et al*. 2004; *Dianthus broteri*, Balao *et al*. 2009; *Ulmus americana*, Whittemore and Olsen 2011; *Aster amellus*, Castro *et al*. 2012; *Allium oleraceum*, Duchoslav *et al*. 2013; *Oxalis obtusa*, Krejčíková *et al*. 2013).

Recent genomic investigations indicate that polyploidy is common in angiosperm lineages including the legume family, and it is postulated that ancient genome-doubling events may be associated with the rapid diversification of this family (Soltis *et al*. 2009; Cannon *et al*. 2010). Legumes are reported to have undergone rapid family-diversification since the Tertiary, which corresponded, for instance, with polyploidization within Papilionoideae (Cannon *et al*. 2010), and emergence of many species as recently as the Plio-Pleistocene (Richardson *et al*. 2001; Lavin *et al*. 2005). In Africa, acacias (*Acacia* Miller s.l., Mimosoideae) diversified during the Pliocene, colonized, expanded and became integral to open, arid-adapted vegetation—the savanna woodlands (Bouchenak-Khelladi *et al*. 2010; Odee *et al*. 2012). Globally, the genus *Acacia* includes >1400 species, and is an important phytogeographic component of the tropics (Lewis *et al*. 2005). However, genome sizes (2C DNA contents) have been explored in only a few species (Bennett and Leitch 2012).

**Figure 1.**
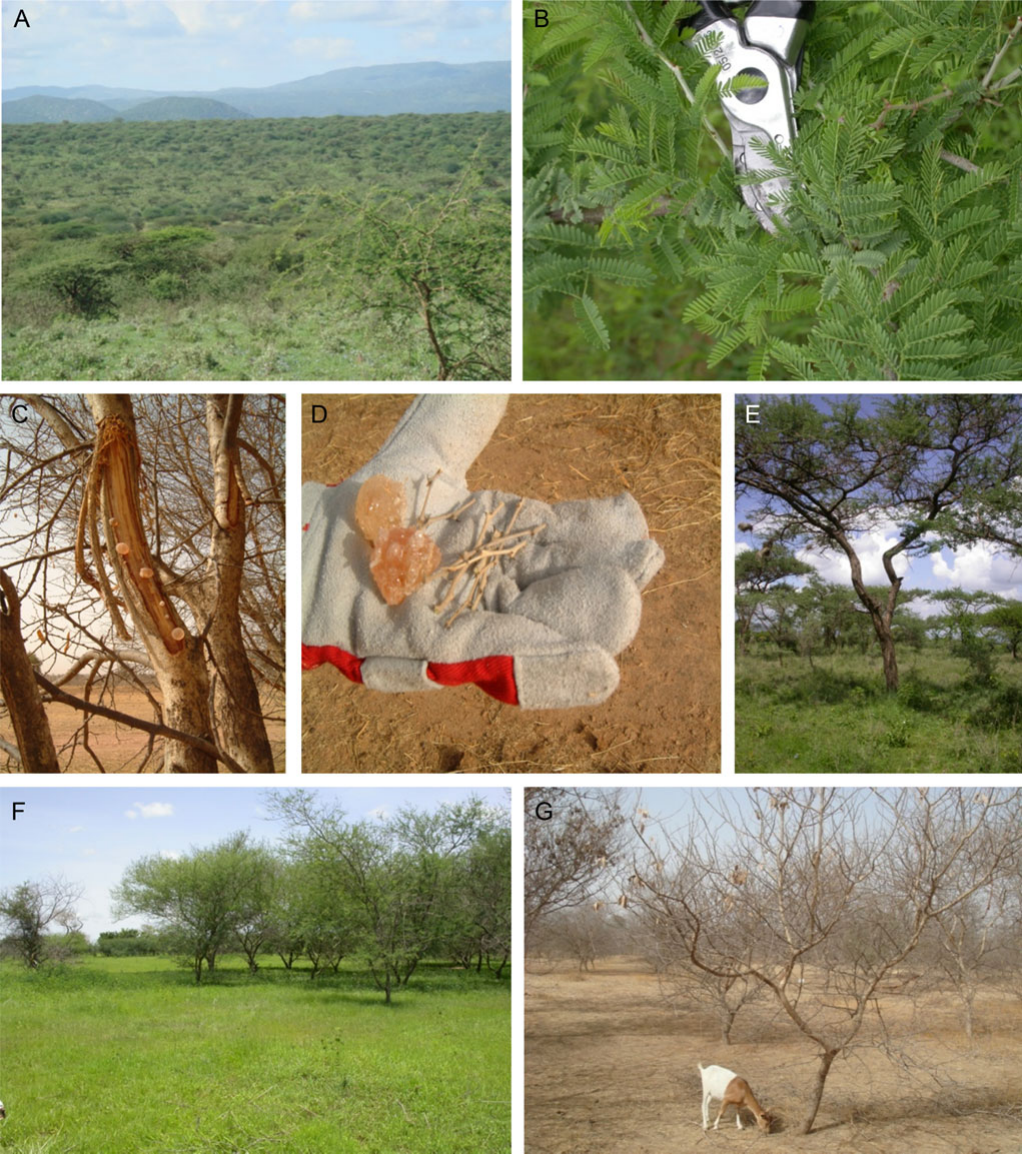
The *Acacia senegal* species: (A) an *A. senegal* woodland savanna during the rainy season, Ngarendare, Kenya; (B) characteristic alternate bipinnate leaves of *A. senegal* used in FCM analysis as fresh or desiccated tissue, a pair of secateurs is placed for scale; (C) gum arabic ‘nodules’ formed on a leafless branch of an *A. senegal* tree 2 weeks after tapping (debarking) at the beginning of dry season at Dahra experimental field trial, Senegal; (D) shows gum ‘nodules’ and twigs on protective glove, they were collected from leafless gum-producing trees, the twigs were stored in silica gel and used in FCM analysis; (E) *A. senegal* tree in Ntumburi, near Isiolo, Kenya; (F) *A. senegal* trees during rainy and (G) dry season at Dahra, Senegal, also showing a goat feeding on fallen pods and seeds.

In this study, we focus on *Acacia senegal* (see Fig. 1), an insect-pollinated outbreeding species, commonly known as the ‘gum arabic’ tree, comprising four recognized varieties based on morphological delimitations, namely vars. *senegal*, *kerensis*, *rostrata* and *leiorhachis* (Fagg and Allison 2004). Together with ∼20 other rare and closely related species, e.g. *A. dudgeoni*, and the diploid *A. asak* as reported by Bukhari (1997*b*), these form the *A. senegal* complex (Ross 1975). *Acacia senegal* is a shrub or tree with considerable economic and ecological importance, producing a natural gum (gum arabic) widely used in the food and beverage industry, pharmaceuticals, other technical applications and provisioning of several ecosystem services in the drylands of tropical Africa (Fagg and Allison 2004; Omondi *et al*. 2010; Sprent *et al*. 2010; Odee *et al*. 2011; Gray *et al*. 2013). Previous cytological studies of *A. senegal* have reported only diploids (Atchison 1948; Oballa and Olng'otie 1993; Bukhari 1997*b*, *c*), but tetraploids were recently detected in three populations in the Sudano-Sahelian region (Assoumane *et al*. 2013), which suggested polyploidy might be prevalent in this region and elsewhere across the native range. However, cytogeographic studies to date have not extensively sampled local and regional spatial scales, nor have populations in the southern native range been examined.

A recent phylogeographic and phylogenetic study by Odee *et al*. (2012) based on ITS and chloroplast DNA (cpDNA) data showed geographic structuring of genetic variation, which separated east and southern African populations (the southern range of the species) from those in west and central Africa within the Sudano-Sahelian region (the northern range). It further inferred that hybridization and introgression were likely to have driven the recent colonization and range expansion of the species in the northern ranges. If the generally held view that polyploidy may confer broader ecological tolerance and colonization ability is true for this species, then we would expect to detect differences in ploidy diversity and distribution within and among populations and regions.

Therefore, we employed flow cytometry (FCM) to survey and assess the rangewide distribution patterns of DNA contents and ploidy levels of *A. senegal*, and specifically addressed the following questions: (i) What is the variation in overall (2C DNA content) and monoploid (1Cx DNA content) genome size? (ii) What is the ploidy variation and distribution pattern across its native range? (iii) Are the ploidy levels phylogeographically and phylogenetically structured? The ploidy data are discussed in the context of origins, evolutionary and recent colonization history of the species. Furthermore, due to difficulties associated with collecting fresh leaf samples in rangewide studies conducted remote from appropriate facilities, we tested a range of tissues, namely seed, fresh leaf and twig, dried leaf and twig and herbarium specimens to evaluate their utility in FCM analysis.

## Methods

### Plant material

Samples were collected between May 2006 and June 2011 from a total of 157 *A. senegal* individuals in wild populations, experimental trials and herbarium specimens representing 54 localities across its natural range in sub-Saharan Africa, Pakistan and India (Table 1). Samples were also obtained from Kadiogo, Burkina Faso for *A. dudgeoni*, a member of the *A. senegal* complex. Samples of different tissues (seed, leaf or twigs) and ages including herbarium specimens that had been stored for >53 months since collection were used for 2C and 1Cx DNA content analysis **[see Supporting Information—Table S1]**. During field collections, fresh terminal twig (∼2–4 mm diameter) or leaf (where available) tissue samples were collected (see Fig. 1D), dried on silica gel in sealable bags, transported back to the lab and stored at 4 °C until used.

**Table 1.** Locations of all sampled populations of *Acacia senegal*, and DNA contents and ploidy levels determined by FCM. 2C DNA values are presented by the mean of all individuals at each population (*n*, number of individuals) ± standard deviation (Mean ± s.d.). Populations represented by a single individual are denoted by n/a (not applicable), in the 2C DNA range (min-max) column. Materials originated from field trials, FT; natural fields, NF; procured seeds (PS) and herbarium specimens (H). Chromosome counts determined in populations marked by single (*), this study, and double (**), previous study (Assoumane *et al*. 2013).

Region Country Population	Variety	Latitude	Longitude	*n*	2C DNA content (pg)	2C DNA content (pg)	DNA ploidy level	1Cx DNA (pg)	Material (origin)	Standard
					Mean ± s.d	Min-max				
East Africa										
Ethiopia										
Sodera**	Senegal	08°24′00.0″N	39°23′00.0″E	5	2.93 ± 0.03	2.90–2.96	4*x*	0.73	Silica-dried twigs (FT)	Solanum
Kenya										
Kakuma	Kerensis	03°45′26.1″N	34°39′59.5″E	2	1.61 ± 0.02	1.59–1.62	2*x*	0.81	Silica-dried leaves (NF)	Solanum
Kaleing’	Kerensis	04°24′28.0″N	35°31′03.1″E	2	1.52 ± 0.09	1.45–1.58	2*x*	0.76	Silica-dried leaves (NF)	Solanum
Kibwezi*	Kerensis	02°12′49.1″S	38°04′22.4″E	3	1.40 ± 0.04	1.37–1.44	2*x*	0.70	Fresh leaves (NF)	Solanum
Kimalel	Kerensis	00°28′17.3″N	35°55′07.9″E	4	1.49 ± 0.05	1.44–1.56	2*x*	0.75	Silica-dried leaves (NF)	Glycine
Kimalel/Sorit	Kerensis	00°28′13.2″N	35°54′32.0″E	5	1.50 ± 0.05	1.44–1.54	2*x*	0.75	Silica-dried leaves (NF)	Glycine
Sorit	Senegal	00°25′39.0″N	35°53′05.0″E	3	1.53 ± 0.02	1.51–1.55	2*x*	0.77	Silica-dried leaves (NF)	Glycine
Kulamawe	Leiorhachis	00°32′21.5″N	37°59′41.5″E	3	1.41 ± 0.04	1.38–1.46	2*x*	0.71	Fresh leaves (NF)	Solanum
Magadi	Leiorhachis	01°31′52.8″S	36°34′31.5″E	2	1.45 ± 0.06	1.41–1.49	2*x*	0.73	Silica-dried leaves (NF)	Solanum
Merille	Kerensis	01°31′40.8″N	37°45′23.7″E	1	1.59	n/a	2*x*	0.80	Silica-dried leaves (NF)	Solanum
Ngarendare	Kerensis	00°28′04.3″N	37°25′02.4″E	2	1.43 ± 0.01	1.42–1.44	2*x*	0.72	Fresh leaves (NF)	Solanum
Ntumburi	Senegal	00°11′54.7″N	37°31′00.2″E	3	2.86 ± 0.06	2.82–2.90	4*x*	0.72	Fresh leaves (NF)	Solanum
Tanzania										
Kigwe	Leiorhachis	06°09′00.0″S	35°21′00.0″E	3	1.29 ± 0.05	1.25–1.34	2*x*	0.65	Fresh leaves (PS)	Solanum
Wangingombe	Senegal	08°30′36.0″S	34°22′48.0″E	2	1.29 ± 0.05	1.25–1.32	2*x*	0.65	Fresh leaves (PS)	Solanum
Sudan										
Kordofan	Senegal	12°44′00.0″N	29°35′00.0″E	5	1.48 ± 0.03	1.44–1.50	2*x*	0.74	Silica-dried twigs (FT)	Solanum
Fallatu Forest*	Senegal	13°06′00.0″N	30°08′24.0″E	4	1.34 ± 0.03	1.30–1.37	2*x*	0.67	Fresh leaves (PS)	Solanum
Central Africa										
Chad										
Chari Baguirmi	Senegal	11°16′26.0″N	16°09′44.0″E	1	2.87	n/a	4*x*	0.72	Fresh leaves (PS)	Solanum
Tourba**	Senegal	12°49′00.0″N	15°18′00.0″E	5	1.57 ± 0.06	1.48–1.64	2*x*	0.79	Silica-dried twigs (FT)	Solanum
Cameroon										
Maroua	Senegal	10°15′00.0″N	14°14′00.0″E	2	2.12 ± 0.02	2.10–2.12	3*x*	0.71	Seed (PS)	Solanum
West Africa										
Burkina Faso										
Bissiga	Senegal	12°26′00.0″N	00°32′00.0″W	7	1.43 ± 0.08	1.30–1.51	2*x*	0.72	Silica-dried twigs/Fresh leaves/seed (FT)	Solanum
Dara	Senegal	14°19′48.0″N	00°10′48.0″W	2	1.43 ± 0.09	1.36–1.49	2*x*	0.72	Fresh leaves/seed (PS)	Solanum
Di	Senegal	13°10′00.0″N	03°25′00.0″W	5	1.49 ± 0.03	1.45–1.53	2*x*	0.75	Silica-dried twigs (FT)	Solanum
Kadiogo	Dudgeoni	12°20′47.0″N	01°26′39.0″W	3	1.38 ± 0.01	1.37–1.38	2*x*	0.69	Fresh leaves (PS)	Solanum
Kiembara	Senegal	13°15′00.0″N	02° 43′12.0″W	1	1.37	n/a	2*x*	0.69	Fresh leaves (PS)	Solanum
Kirbou	Senegal	13°15′36.0″N	02°04′ 12.0″W	2	1.32 ± 0.05	1.28–1.35	2*x*	0.66	Fresh leaves/twigs	Solanum
Kantchari	Senegal	12°22′48.0″N	01°30′00.0″E	1	1.34	n/a	2*x*	0.67	Seed (PS)	Solanum
Mali										
Aite	Senegal	15°05′00.0″N	11°39′00.0″W	4	1.45 ± 0.07	1.38–1.53	2*x*	0.73	Silica-dried twigs (FT)	Solanum
	Senegal	15°05′00.0″N	11°39′00.0″W	1	2.80	n/a	4*x*	0.70	Silica-dried twigs (FT)	Solanum
Kirane	Senegal	15°23′00.0″N	10°15′00.0″W	4	2.87 ± 0.11	2.71–2.97	4*x*	0.72	Silica-dried twigs (FT)	Solanum
Somo**	Senegal	13°17′00.0″N	04°54′00.0″W	4	1.45 ± 0.05	1.37–1.48	2*x*	0.73	Silica-dried twigs (FT)	Solanum
Mauritania										
Djiguéni	Senegal	15°44′00.0″N	08°40′00.0″W	5	1.47 ± 0.04	1.42–1.53	2*x*	0.74	Silica-dried twigs (FT)	Solanum
Kankossa	Senegal	15°56′00.0″N	11°27′00.0″W	5	1.46 ± 0.06	1.37–1.53	2*x*	0.73	Silica-dried twigs (FT)	Solanum
Niger										
Karofane	Senegal	14°18′00.0″N	06°11′00.0″E	4	1.52 ± 0.06	1.47–1.60	2*x*	0.76	Silica-dried twigs (FT)	Solanum
	Senegal	14°18′00.0″N	06°11′00.0″E	1	2.13	n/a	3*x*	0.71	Silica-dried twigs (FT)	Solanum
Senegal										
Daiba	Senegal	15°22′00.0″N	13°08′00.0″W	5	1.44 ± 0.06	1.37–1.49	2*x*	0.71	Silica-dried twigs (FT)	Solanum
Diamenar	Senegal	16°00′00.0″N	15°54′00.0″W	5	1.47 ± 0.03	1.44–1.50	2*x*	0.74	Silica-dried twigs (FT)	Solanum
Kidira**	Senegal	14°28′00.0″N	12°13′00.0″W	5	1.50 ± 0.06	1.41–1.58	2*x*	0.75	Silica-dried twigs (FT)	Solanum
	Senegal	14°28′00.0″N	12°13′00.0″W	1	4.51	n/a	6*x*	0.75	Silica-dried twigs (FT)	Solanum
Ngane	Senegal	14°08′00.0″N	16°12′00.0″W	3	1.48 ± 0.08	1.40–1.55	2*x*	0.74	Silica-dried twigs (FT)	Solanum
	Senegal	14°08′00.0″N	16°12′00.0″W	2	2.90 ± 0.03	2.88–2.92	4*x*	0.73	Silica-dried twigs (FT)	Solanum
Ranerou	Senegal	15°17′45.2″N	13°57′18.7″W	4	2.72 ± 0.03	2.69–2.75	4*x*	0.68	Silica-dried leaves (PS)	Solanum
Southern Africa										
Limpopo	Leiorhachis	22°25′48.5″S	31°11′04.0″E	1	1.59	n/a	2*x*	0.80	Silica-dried leaves (H)	Solanum
Mpumalanga_1	Rostrata	25°28′05.4″S	31°30′02.6″E	1	1.59	n/a	2*x*	0.80	Silica-dried leaves (H)	Solanum
Mpumalanga_2	Rostrata	25°15′12.8″S	31°54′11.5″E	1	1.61	n/a	2*x*	0.81	Silica-dried leaves (H)	Solanum
Mpumalanga_3	Rostrata	25°20′22.4″S	31°44′07.9″E	1	1.57	n/a	2*x*	0.79	Silica-dried leaves (H)	Solanum
Mpumalanga_4	Rostrata	24°59′47.6″S	31°35′08.8″E	1	1.57	n/a	2*x*	0.79	Silica-dried leaves (H)	Solanum
Mpumalanga_5	Rostrata	24°45′49.2″S	31°41′40.1″E	1	1.54	n/a	2*x*	0.77	Silica-dried leaves (H)	Solanum
Mpumalanga_6	Rostrata	24°26′01.6″S	31°32′45.1″E	1	1.61	n/a	2*x*	0.81	Silica-dried leaves (H)	Solanum
Mpumalanga_7	Leiorhachis	24°05′54.8″S	31°41′31.5″E	1	1.51	n/a	2*x*	0.76	Silica-dried leaves (H)	Solanum
Mpumalanga_8	Leiorhachis	24°05′50.7″S	31°41′31.9″E	1	1.57	n/a	2*x*	0.79	Silica-dried leaves (H)	Solanum
Mpumalanga_9	Leiorhachis	24°04′06.6″S	31°41′20.0″E	1	1.53	n/a	2*x*	0.77	Silica-dried leaves (H)	Solanum
Mpumalanga_10	Leiorhachis	25°26′46.9″S	30°58′06.4″E	1	1.51	n/a	2*x*	0.76	Silica-dried leaves (H)	Solanum
Mpumalanga_11	Rostrata	25°26′46.9″S	30°58′06.4″E	1	1.57	n/a	2*x*	0.79	Silica-dried leaves (H)	Solanum
Namibia										
Kunene	Rostrata	17°01′15.7″S	13°09′40.8″E	1	1.58	n/a	2*x*	0.79	Silica-dried leaves (H)	Solanum
Pakistan and India										
India										
Jodhpur-Inde50/60	Senegal	26°19′00.0″N	79°31′00.0″E	3	1.51 ± 0.08	1.44–1.59	2*x*	0.76	Silica-dried twigs (FT)	Solanum
Jodhpur-Inde50/60	Senegal	26°19′00.0″N	79°31′00.0″E	6	3.00 ± 0.13	2.73–3.09	4*x*	0.75	Silica-dried twigs (FT)	Solanum
Jodhpur	Senegal	26°42′54.4″N	73°08′31.4″E	2	1.35 ± 0	n/a	2*x*	0.68	Fresh leaves (PS)	Solanum
Pakistan										
Sind, Off Thano-Bula-Kotri Rd	Senegal	25°19′28.9″N	68°03′58.9″E	1	2.89	n/a	4*x*	0.72	Silica-dried leaves (H)	Solanum
Sind, Off Budhapur-Menjhand Rd	Senegal	25°41′23.4″N	68°19′01.5″E	1	2.99	n/a	4*x*	0.75	Silica-dried leaves (H)	Solanum

### Chromosome counts

Chromosome numbers were determined from mitotically active root tip meristems of seedlings germinated from seeds from two populations (Kibwezi and Fallatu, Table 1) using standard methods described for acacias (Bukhari 1997*a*). Chromosome counts for other populations, namely Kidira, Somo and Tourba; and Sodera, representing both diploids and tetraploids have previously been determined (Assoumane *et al*. 2013). Chromosome counts in other populations from previous studies are presented **[see Supporting Information—Table S2]**.

### Flow cytometry, determination of DNA content and ploidy levels

All 157 individuals were subjected to DNA content and ploidy-level estimation using FCM. Sample preparation followed the one-step protocol of Doležel *et al*. (2007), using an internal plant standard and propidium iodide (PI) dye, which is a DNA selective fluorochrome with no base specificity, and Tris · MgCl_2_ buffer (Doležel *et al*. 2007). For each sample, 1 mL of chilled buffer was placed in a small (5 cm diameter) Petri dish, on ice. Twigs were wiped with damp tissue to remove surface debris, and gently scraped with a razor blade, removing tangential slivers of the outer layers of the tissues, up to and including the green cambial layer, and the scrapings (4 mg) were collected into the buffer. For leaf samples, 4 mg of fresh or dried tissue of the test plant were used. Fresh leaf tissue (25 mg) of a DNA primary reference standard of glasshouse-grown tomato (*Solanum lycopersicum* ‘Stupické polní rané’, 2C = 1.96 pg DNA), soya bean (*Glycine max* Merr. ‘Polanka’, 2C = 2.50 pg DNA) or maize (*Zea mays* ‘CE-777, 2C = 5.43 pg DNA) plants (Doležel *et al*. 2007) were chopped together with the test samples with a new feather-edge razor blade, cutting pieces measuring 0.5–2 mm across (taking ∼60–90 s). For seeds, radicle tissue was used as described by Śliwińska *et al*. (2005) to avoid endoreduplicated cells in endosperms. The resultant suspension was collected into a 1 mL pipette and filtered through squares of 42-µm nylon mesh (Sefar AG, Switzerland) into standard 5-mL flow cytometer collection tubes, on ice. RNase was added to prevent staining of double-stranded RNA, followed by PI to attain final concentrations of 80 µg mL^−1^ for both. Samples were then briefly and gently vortexed to mix, and incubated on ice in the dark until they were measured (usually within 10–50 min).

The use of different tissues was tested in a range of studies, including comparisons of data collected from fresh leaves and twigs from the same plant, and dried leaves and stems from the same plant. Analyses were repeated on several samples to determine the reliability of the data obtained.

Samples were analysed with a Becton Dickinson FACSCalibur flow cytometer (San Jose, CA, USA), equipped with an argon-ion 488-nm wavelength laser, calibrated daily and run on the low flow rate. Counts were collected using the FL2 (orange) detector with a band-pass filter of 585/42 nm. The default threshold setting on FL2 was used (channel 52). Instrument settings (voltage and gain) were adjusted to allow for fluorescence intensity of expected ploidy levels, together with the peak of the reference plant, to be located between channels 250–750 on the *x*-axis (using a 1024 scale) of plots of counts of nuclei vs FL2A. Forward scatter (FSC) and side scatter (SSC) were set to logarithmic scales, and FL2 was set to linear.

Data were acquired with CellQuest software (v4.0.2; Becton Dickinson) and subsequently analysed using Cyflogic™ software (version 1.2.1; Cyflo Ltd, Finland), determining the DNA content of the test sample against that of the internal reference standard. Gating of the populations of interest was done first on the FSC/SSC dot plot, and then on the FL2A (area) vs FL2W (width) dot plot to remove debris and doublets, and histograms of a number of nuclei vs channel number (relative fluorescence) were then plotted. The peak mean channel number and full peak coefficients of variation (CV) of each peak were determined. DNA contents of the samples were calculated from the ratios of the peak mean channel numbers of the test and reference plant material. The samples were assigned to ploidy levels based on the available chromosome counts and their DNA contents.

### Statistical analysis

**Figure 2.**
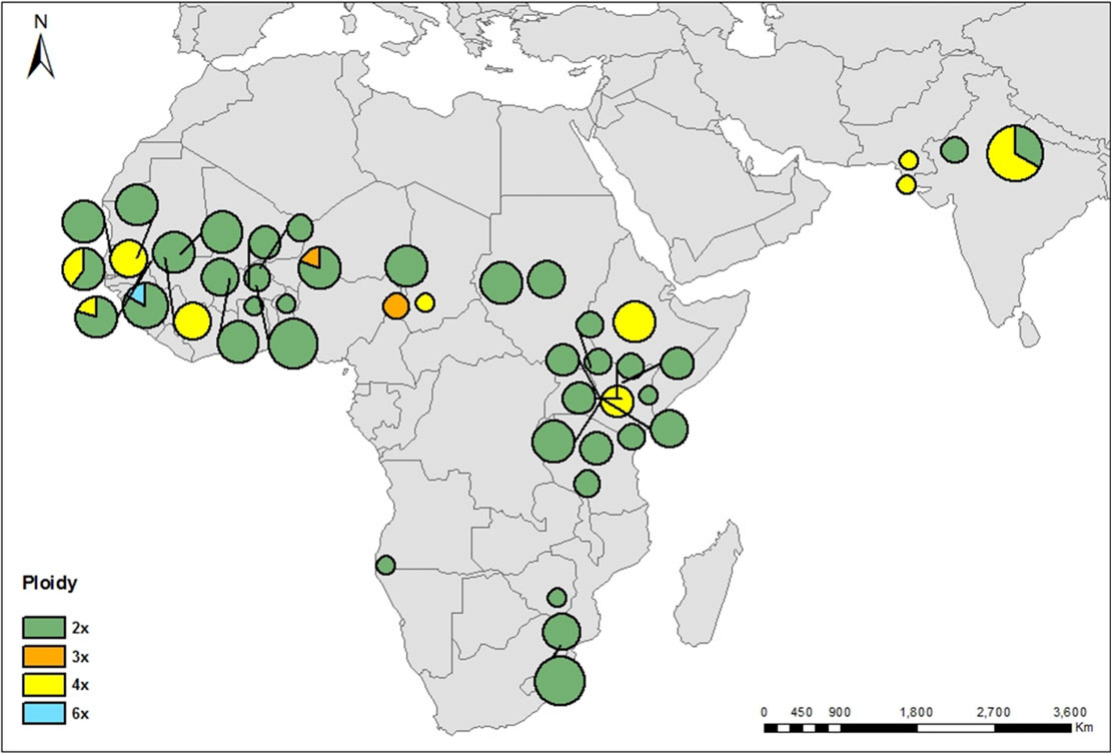
Rangewide distribution of *A. senegal* ploidy levels identified by colour (see legend). Circle area and pie slice represent sample size and relative frequency. Dark lines indicate precise geographic location of the populations.

**Figure 3.**
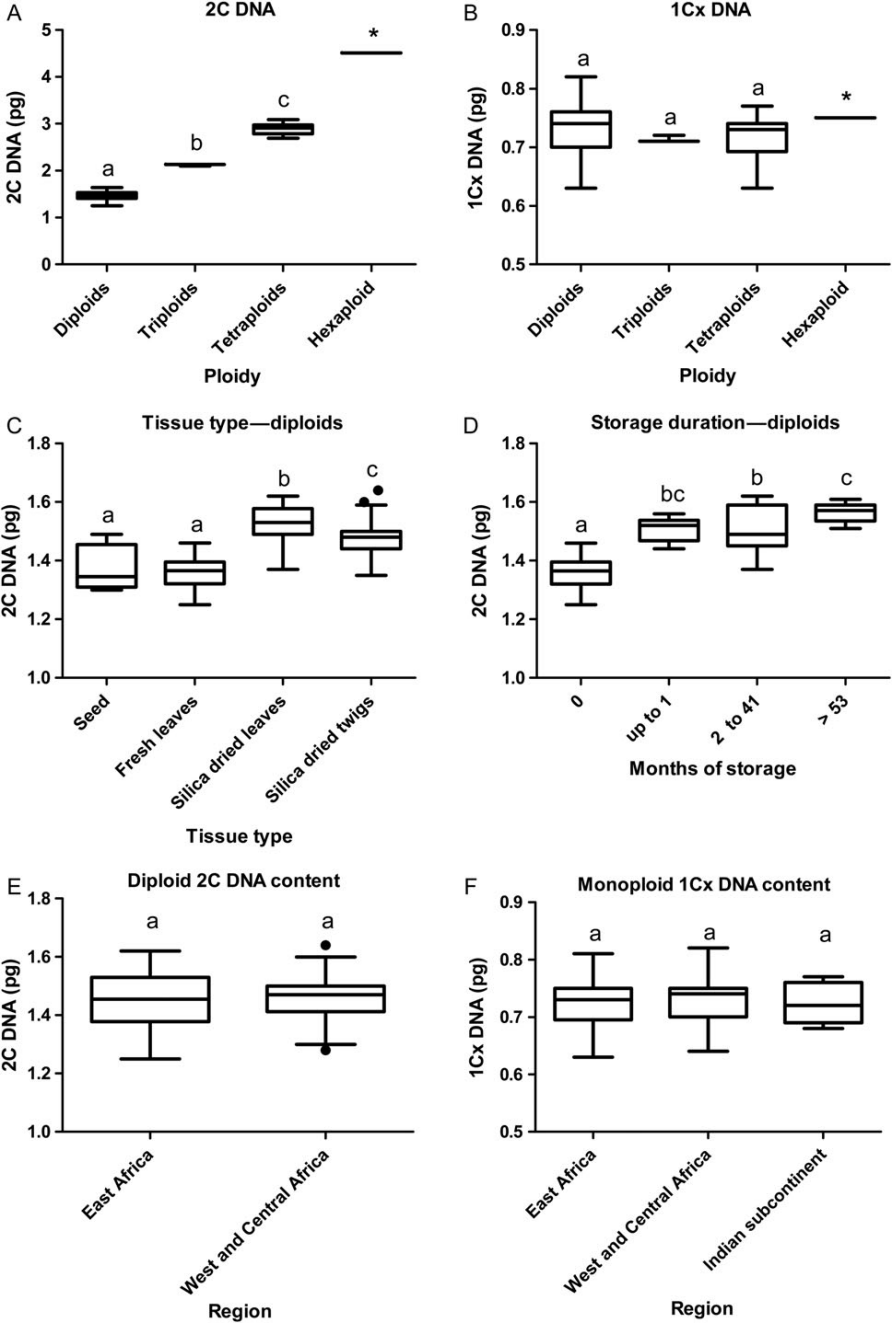
Box-plot representations of *A. senegal* (A) 2C DNA, (B) monoploid (1Cx) values comparing ploidy levels; (C) 2C DNA comparing tissue types of diploid individuals, (D) 2C DNA values comparing storage duration; (E) 2C DNA of diploid individuals and (F) monoploid (1Cx) values of all ploidy levels comparing biogeographical regions. Means not significantly different at *P* < 0.05 are indicated by the same letter (Tukey's post test, or two-tailed unpaired *t*-test). Horizontal lines represent the median, and boxes and whiskers, respectively, the interquartile range and the non-outlier ranges. Circles denote outliers. An asterisk (*) indicates the single hexaploid DNA content value (4.51 pg) is not included in the ANOVA analysis.

The 2C DNA content was calculated for each sample as the average of the replicates, and monoploid genome size (1Cx) estimated as the amount of nuclear DNA divided by ploidy level (Table 1). Either one-way ANOVA followed by Tukey's multiple comparison tests or two-tailed unpaired *t*-test were performed with 2C DNA and 1Cx DNA contents as the dependent variables. Analyses comprised the variation in 2C DNA and 1Cx DNA contents with ploidy (Fig. 3A and B); the effects of tissue type (seed, leaf and dried leaf and twigs) on diploid 2C DNA content (Fig. 3C); and the effects of storage duration (arbitrarily categorized as 0, between 2 and 41 and >53 months) on 2C DNA content (Fig. 3D). Regional comparisons were carried out among populations from east Africa, west and central Africa and the Indian subcontinent (India and Pakistan) using only diploid individuals for 2C DNA (Fig. 3E) and all ploidy levels and individuals for 1Cx DNA contents (Fig. 3F). Southern Africa was excluded from these regional analyses because possible regional variation could not be separated from variations due to tissue storage duration. All analyses and graphs were performed with GraphPad Prism version 5.04 for Windows, GraphPad Software, La Jolla, CA, USA, www.graphpad.com. The DNA ploidy distribution was mapped using the ESRI software ArcMap 10.1 (ESRI, Redlands, CA, USA) (Fig. 2). Eleven herbarium specimens collected from Mpamalanga province (Republic of South Africa) were grouped and mapped into two geographically proximate quasi populations of four and seven individuals, taxonomically affiliated to vars. *leiorhachis* and *rostrata*, respectively (Table 1) **[see Supporting Information—Table S1]**.

**Figure 4.**
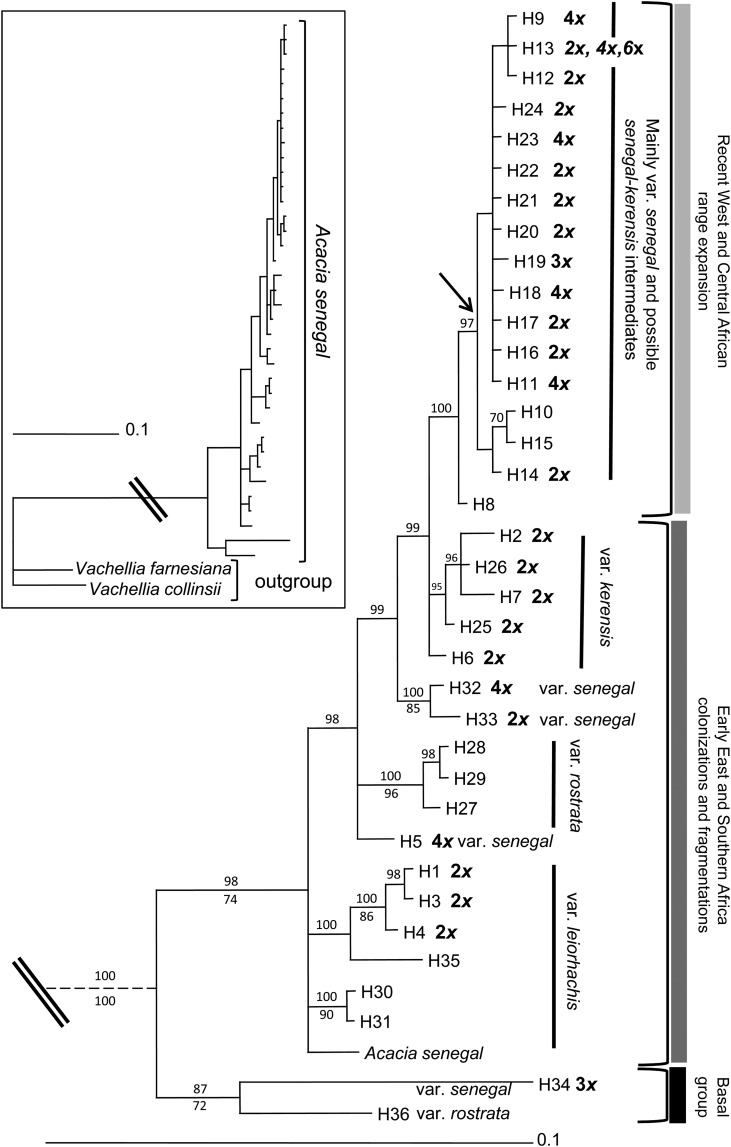
Bayesian 50 % majority-rule consensus phylogram of ITS sequences (GenBank accession numbers HQ605042–HQ605077) from *Acacia senegal* individuals sampled across its distribution range (adapted from Odee *et al*. 2012). Each haplotype is labelled by H and the number (1–36), followed by the DNA ploidy indicated in bold where analysed. Branches are labelled with ≥70 % bootstrap support (below) and posterior probability (above) values. Arrow indicates presence of a single clade in the maximum parsimony strict consensus tree. The tree was rooted with *Acacia* (syn. *Vachellia*) *farnesiana* and *Acacia* (syn. *Vachellia*) *collinsii* (see inset tree, double line denotes the point at which the break is in the main tree) obtained via GenBank (accession numbers EF638219 and EF638216, respectively). *Acacia senegal* sequence is accession number EF638213 from Zimbabwe (ploidy unknown). Clades are identified by vertical (dark) lines, labelled with constituent *A. senegal* varieties (*senegal*, *kerensis*, *rostrata* and *leiorhachis*). Hypothesized recent range expansion and early colonization and fragmentation events are indicated by light and dark grey vertical bars. Scale bar signifies 0.1 substitutions per nucleotide site.

The DNA ploidy data of analysed individuals were also located on the phylogenetic tree (maximum parsimony strict consensus tree) of *A. senegal* based on ITS sequence data (Fig. 4) (Odee *et al*. 2012). Examples of FCM histograms collected from different tissue types are provided in **Supporting Information—Fig. S1**.

## Results

### Chromosome counts

Chromosome numbers for Kibwezi (*A. senegal* var. *kerensis*) and Fallatu (*A. senegal* var. *senegal*) populations were 2*n* = 26, corresponding to diploid.

### Flow cytometry measurements and effects of tissue types and storage

Flow cytometry analyses generally produced clear peaks **[see Supporting Information—Fig. S1]** and output from fresh or dry twigs **[see Supporting Information—Fig. S1A and C]** was of similar quality to that of comparable leaf samples **[see Supporting Information—Fig. S1B and D]**. When fresh leaf and twig were combined in the same extract **[see Supporting Information—Fig. S1E]**, the peak positions were identical. The dried leaf showed a slightly higher peak position than the dried stem **[see Supporting Information—Fig. S1D vs C]**, as is also indicated in Fig. 3C. Overall, the mean full peak CVs of standards ranged from 1.78 to 2.89 %, while those of samples (including dried leaf and twigs stored 2–41 months) ranged from 1.76 to 5.09 % **[see Supporting Information—Table S3]**. The CVs of samples from southern Africa, which had been stored for >53 months ranged from 4.38 to 7.57 %. Fresh material (leaves and seed) generally yielded lower CVs than dried samples. The mean 2C DNA contents of diploids, triploids and tetraploids were significantly different (Fig. 3A). The DNA content of the hexaploid was not included in the statistical analysis but was 3.06-fold higher than the corresponding diploid DNA content. The monoploid DNA content across all the ploidy series ranged from 0.63 to 0.81 pg (Table 1), but the mean monoploid DNA contents among the diploids, triploids and tetraploids were not significantly different (Fig. 3B). Analysis of variance results showed that the 2C DNA value of diploid individuals was dependent on tissue type (seed, fresh leaves, dried leaves and twigs) (*F*_3,121_ = 40.8, *P* < 0.0001); seed and fresh leaves were not significantly different according to Tukey's post test (Fig. 3C), and were lower than dried leaves and twigs. There was also a significant effect (*F*_3,56_ = 46.7, *P* < 0.0001) of storage (period between sample collection and FCM analysis) (Fig. 3D). No significant differences between regions were detected in the comparisons of 2C DNA of diploids (Fig. 3E) and 1Cx DNA (Fig. 3F).

### DNA content variation and ploidy levels

The 2C DNA contents of all 157 individuals ranged from 1.25 to 4.51 pg (Table 1) **[see Supporting Information, Table S1]**. Taking both fresh and dried *A. senegal* tissue samples into consideration, we found mean 2C DNA (pg ± s.d.) contents of 1.47 ± 0.09 (*n* = 126), 2.12 ± 0.02 (*n* = 3), 2.89 ± 0.12 (*n* = 28), and a single individual with 4.51 pg, corresponding to a polyploid series of diploid, triploid, tetraploid and hexaploid (Table 1) **[see Supporting Information, Table S1]**. The mean 2C DNA contents of fresh samples from Kibwezi and Fallatu, 1.40 ± 0.04 pg (*n* = 3) and 1.34 ± 0.03 pg (*n* = 4), respectively, were confirmed as diploids by chromosome counts (2*n* = 2*x* = 26). The Sodera population had a mean 2C DNA value of 2.93 ± 0.03 pg (*n* = 5; Table 1) and was recently confirmed as tetraploid with chromosome counts (2*n* = 4*x* = 52) and microsatellite markers (Assoumane *et al*. 2013). In essence, 2C DNA values for the predominant diploids varied from 1.25 to 1.64 pg (1.31-fold variation, *n* = 126), while tetraploids ranged from 2.69 to 3.09 pg (1.15-fold, *n* = 28), and the rare triploids, 2.13 to 2.17 pg (1.02-fold, *n* = 3). Most populations (90.7 %) were of single ploidy level (i.e. uniform ploidy; 2*x*, 3*x* or 4*x*), while five populations (9.3 %) presented mixed ploidy levels as follows: diploid and tetraploid (Aite, Ngane and Jodhpur), diploid and triploid (Karofane) and diploid and hexaploid (Kidira), all of which were in the Sudano-Sahelian and Indian subcontinent regions (Table 1, Fig. 2) **[see Supporting Information, Table S1]**. Triploid and hexaploid individuals were rare. The triploid individuals occurred in Maroua, Cameroon and Karofane, Niger (Fig. 2, Table 1). Both triploids and the hexaploid detected by FCM are reported for the first time, but have not been confirmed by chromosome counts due to their rarity.

### Cytogeography

The overall distribution pattern of ploidy levels is shown in Fig. 2 (Table 1) **[see Supporting Information—Table S2** with additional data from previous studies (Bukhari 1997*b*, *c*; Assoumane *et al*. 2013)**]**. Diploids were the most common and widespread, occurring in the entire natural geographic range of the species. *Acacia dudgeoni* (analysed in this study) and *A. asak* (previously reported by Bukhari (1997*b*)) are also diploids **[see Supporting Information—Table S2]**. Tetraploids, the rare triploids and hexaploid individuals occur in the Sudano-Sahelian region. Only two tetraploid populations occur in the east African Highlands (Sodera, Ethiopia, and Ntumburi, Kenya), while populations south of the Equator were all diploids. Both diploids and tetraploids were also detected in populations from Pakistan and India in spite of limited sampling of these regions. Interestingly, all detected polyploids belonged to *A. senegal* var. *senegal*, although diploids were still the majority in this taxon, and none were detected in the vars. *kerensis*, *leorhachis*, *rostrata* or *A. dudgeoni*.

### Phylogeny and phylogeography of *A. senegal* diploids and polyploids

The ITS and cpDNA haplotypes were variably shared within and among diploids and polyploids **[see Supporting Information—Table S1]**. The diploid–polyploid complexes occurred mostly in the terminal clade, which comprised mainly Sudano-Sahelian samples affiliated to var. *senegal* (Fig. 4). Diploids dominated the basal clades, which largely comprised east and southern Africa populations (southern range), and consisted of individuals affiliated to either var. *leiorhachis* or vars. *senegal*, *kerensis* and *rostrata*.

## Discussion

### Flow cytometry: methodological limitations and innovations

Our main objective to survey and assess the rangewide distribution patterns of DNA contents and ploidy levels of *A. senegal* was dependent on the utility and reliability of the FCM methodological innovations we developed in order to circumvent the difficulties associated with collecting fresh leaf samples in the species’ native range. While other studies have tested the use of herbarium leaf samples, our use of the outer tissues of dried twigs is novel. Flow cytometry comparison of fresh leaf and twig and dried leaf and twig indicated that they yielded similar results **[see Supporting Information—Fig. S1]**. Both dried leaves and dried twigs showed elevated 2C DNA amounts (Fig. 3C) relative to fresh material. Previous studies by Bukhari (1997*c*) of six diploid populations across the northern range of the species (Sudan, Senegal, India and Pakistan) **[see Supporting Information—Table S2]** yielded estimates of 1.11 ± 0.09–1.17 ± 0.04 pg (mean ± standard error) with ‘White Leghorn’ chicken (*Gallus domesticus*) erythrocytes as internal standard, compared with our fresh leaf mean 2C DNA content of 1.36 ± 0.06 s.d. pg, *n* = 25. We attribute this difference to the internal standards used, especially as a considerable variation in 2C genome size for ‘Leghorn’ chickens of different strains and sexes has been reported (Mendonça *et al*. 2010). Our data also showed that triploid, tetraploid and hexaploid individuals had 2C DNA contents commensurate with their ploidy levels. The bulk of plant tissues used in our study were dried twigs. The estimation of DNA content from dried twig and leaf samples mostly yielded results with full peak CVs <5 %, thus falling within the generally accepted upper limit (Doležel *et al*. 2007). The CVs of the fresh reference plants, measured simultaneously with the test species were lower, mostly <3 %. However, CVs of up to 7.57 % were also obtained in some herbarium individuals from the southern African region; these samples were long-stored leaves (up to 58 months) of herbarium specimens. As well as increasing CVs of stored tissues, storage also resulted in increased 2C DNA values; using our fresh leaf mean 2C DNA content for diploids (1.36 ± 0.06 pg) as a benchmark, we observed an increase of 15 % for dried leaf and twig tissues stored >53 months (cf. 1.57 ± 0.07 pg), highlighting the compounding effects of desiccation and storage duration of tissue material. Nonetheless, these variations (between fresh and dried tissues) are within the ranges reported for various other species that have used either dried tissues and/or storage conditions (e.g. Šmarda and Stančík 2006; Suda and Trávníček 2006; Balao *et al*. 2009; Cires *et al*. 2009; Bainard *et al*. 2011). In spite of the variation, we could still unequivocally assign samples to the appropriate ploidy.

The use of the external tissues of dried twigs in FCM analysis of woody plants is new. The only previous FCM use of twigs employed decorticated moist winter twigs of Icelandic birch species (*Betula nana* and *B. pubescens*; Anamthawat-Jónsson *et al*. 2010). The use of dried twigs enables collections in dormant seasons and populations that are situated far from laboratory facilities for FCM determinations. This will open up opportunities for numerous other understudied dryland acacias and woody species.

### DNA ploidy variation, diversity and distribution

To the best of our knowledge, this is the highest polyploid series reported for *A. senegal. Acacia tortilis*, which also has a diploid chromosome count of 2*n* = 2*x* = 26, is reported to be 2*x*, 3*x*, *4x* and 8*x* (e.g. Oballa and Olng'otie 1993; Bukhari 1997*b*, *c*; El Ferchichi Ouarda *et al*. 2009), and the Australian acacia, *A. dealbata* (2*x*, 3*x* and 4*x*, Blakesley *et al*. 2002). However, unlike *A. senegal*, other studied African acacias, namely *A. tortilis* and *A. nilotica*, appear to be predominantly polyploids (Bennett and Leitch 2012). Our results report populations of mixed ploidy in the species for the first time. Rangewide determination of DNA ploidy in *A. senegal* found diploids, triploids, tetraploids and a hexaploid including some populations with multiple ploidy levels (2*x* + 4*x*, 2*x* + 3*x* and 2*x* + 6*x*). Diploids were the most common and widespread, occurring throughout the range (Fig. 2). Tetraploids constituted 17.8 % of the samples analysed, and together with the rare triploids and hexaploid, they were only found in the northern range and only with var. *senegal*, mostly in the Sudano-Sahelian regions of central and west Africa. The east African region, represented by the three common varieties (vars. *senegal*, *kerensis* and *leiorhachis*), had relatively higher sampling intensity than the other regions. In this region, sampled populations immediately south of the Equator were all diploids. Further south (South Africa and Namibia), localities were represented by several single herbarium specimens of vars. *rostrata* and *leiorhachis*, and were also all diploids. Thus, our results suggest that if polyploids are present in the southern range, they are likely to be in low frequencies.

### Origins and evolution of polyploidy in *A. senegal*, and range expansion

The wide spatial distribution of polyploids, coupled with their occurrence at different levels in the phylogenetic tree (Figs 2 and 4) suggests that polyploids have arisen on multiple independent occasions, including contemporary ecological times to the present. The diploid–polyploid complexes shared the major haplotypes indicating gene flow among the different ploidy levels (Fig. 4) **[see Supporting Information—Table S1]**. In particular, the distribution of diploid–polyploid complexes in the terminal clade, comprising mainly the west and central African (Sudano-Sahelian region) populations, is also consistent with the hypothesis of a recent range expansion of the species in that region based on ITS and cpDNA data (Odee *et al*. 2012). Young polyploid complexes are characterized by many diploids and a few tetraploids (Stebbins 1971, cited by Meyers and Levin 2006). In the same Sudano-Sahelian region, Assoumane *et al*. (2013) detected three tetraploid populations not sharing the same chlorotypes, and suggested multiple origins including allopolyploidy. However, the constancy in monoploid DNA content among our diploid–polyploid populations in the Sudano-Sahelian and Indian subcontinent regions also suggests the possibility of autopolyploid origin. Lack of current data on species composition, distribution and ecology, especially of *A. senegal* and potential progenitors of the ploidy series observed in this expansive and ecologically heterogeneous geographical region, does not allow for precise inferences on the origins. The documented localities of early herbarium collections (e.g. Ross 1975, 1979; Fagg and Allison 2004) show extensive distribution of the *A. senegal* complex and other congeneric populations, with similar base chromosome numbers and therefore potential sources for allopolyploidy. Clear evidence of allopolyploidy within acacia is demonstrated by the triploid *Acacia laeta* (2*n* = 39), which is a hybrid between *A. senegal* var. *senegal* and *A. mellifera* subsp. *mellifera* found in the Sudano-Sahelian region, as well as eastern Africa and the Arabian peninsula (Khan 1951, cited by Fagg and Allison 2004; Ross 1979). Our study did not sample any known *A. laeta* population, but we detected triploids only in three individuals found in two *A. senegal* var. *senegal* populations, Maroua and Karofane, with the latter occurring as a minority cytotype within a diploid population (2*x* + 3*x*). The two samples from Maroua were both from seed, which might not have had long-term viability, while the sample from Karofane was taken from a mature tree. These populations with triploid individuals suggest two possible scenarios: the triploids could have arisen either from a diploid × tetraploid cross, or from combination of reduced and unreduced gametes of a diploid (Petit *et al*. 1999). However, there are no known diploid or tetraploid populations in proximity of Maroua population, Cameroun (Fig. 2).

In this study, the Sudano-Sahelian and the Indian subcontinent regions broadly represent the contact zones for diploid–polyploid complexes in *A. senegal*. Notwithstanding the relatively low representation of DNA content and ploidy data from the southern region, it is clearly evident from this study that there is a north–south divide in diploid–polyploid distribution of the species. The tetraploid Ntumburi population, from Kenya (00°11′54.7″N, 37°31′00.2″E), was the southernmost polyploid population; all of the populations south of the equator were diploids. The *Acacia senegal* species complex is young in evolutionary terms predating the Pleistocene (Bouchenak-Khelladi *et al*. 2010; Odee *et al*. 2012), and the terminal clade of the phylogenetic tree (Fig. 4) depicts a relatively recent and rapid expansion into the northern range from eastern Africa (Odee *et al*. 2012). This expansion may have been facilitated by the polyploidization events in the distinctive var. *senegal*, which is drought-tolerant and high gum-producing compared with the other varieties (Fagg and Allison 2004). The association of var. *senegal* with polyploids may also suggest that polyploidization is driving its differentiation from the other varieties. Furthermore, the diploid–polyploid contact zones also appear to coincide with cpDNA haplotype disjunctions located in westernmost part of west Africa (western Senegal) and central Africa, proposed to be refugial zones for *A. senegal* during the climate oscillations of the Plio-Pleistocene (Odee *et al*. 2012).

## Conclusions

Through rangewide analysis of ploidy levels, we have shown in this study that *A. senegal* is predominantly diploid, but has new ploidy levels which we report for the first time. The occurrence of diploid–polyploid complexes in the northern range is congruent with existing phylogenetic and phylogeographic data and supports the hypothesis that polyploidization may have been crucial to the recent colonization and range expansion in these regions. However, further studies that employ greater sampling intensity at various spatial scales, focussing on the contact zones of the Sudano-Sahelian region, as well increasing resolution of southern African populations, will be necessary to detect whether polyploidy exists in localities and regions where it has not been reported. This study demonstrated the use of dried twigs as a good alternative source of leaf tissue for determinations of reliable DNA content and ploidy levels, which provides opportunities for rangewide screening of numerous other understudied dryland species using FCM.

## Sources of Funding

This work was supported by the European Commission, through the ACACIAGUM Project [FP6 Contract No. 032233]. D.W.O. was also supported by a Marie Curie Fellowship [Contract MIIF-CT-2006-39216].

## Contributions by the Authors

D.W.O. and J.W. designed the experiment and performed FCM analysis; all authors participated in sample collection, data analysis and writing of the manuscript. They all have seen and agreed to the submitted manuscript.

## Conflicts of Interest Statement

None declared.

## Supporting Information

The following additional information is available in the online version of this article –

**Table S1**. Locations of sampled *Acacia senegal* populations, DNA content and ploidy levels analysed by FCM, and corresponding nuclear ITS and chloroplast PCR-RFLP *trn*H-*psb*A haplotype data derived from a previous study (Odee *et al*. 2012).

**Table S2.** Species, locations, 2C DNA content and ploidy levels (chromosome counts) of *Acacia senegal* from previous studies.

**Table S3.** 2C DNA content and ploidy of *Acacia senegal* determined from dried twigs, dried leaves and fresh leaves, with *Solanum lycopersicum* ‘Stupické polní rané’ as the internal standard and Tris.MgCl_2_ buffer. Dried twigs had been stored for 41 months and dried leaves for 2 months. Samples measured were each taken from individual plants, not repeat measurements on the same plant or same extract.

**Figure S1.** Number of nuclei vs relative fluorescence intensity (FL2-A 1024 linear channel scale) obtained after simultaneous extraction and analysis of nuclei isolated from (A) fresh twigs and (B) fresh leaves of DNA diploid *Acacia senegal* (Kirbou) (G1 peak) and fresh internal reference standard, *Solanum lycopersicum* ‘Stupické polní rané’ (2C = 1.96 pg DNA), showing G0/G1 and G2/M peaks of the solanum cell cycle), (C) dried twigs and (D) dried leaves of DNA diploid *A. senegal* (Bissiga) and fresh internal solanum reference standard and (E) a combined extract of fresh twig and leaf of DNA diploid *A. senegal* (Fallatu Forest) and fresh internal solanum reference standard. Horizontal bars represent the full peak widths used for determination of full peak coefficients of variation. Solanum and acacia samples A, B and E were from fresh glasshouse grown material, acacia samples C and D had been stored in silica gel for 41 months before extraction and analysis.
